# Breakfast intake and associated factors and barriers among tertiary institution students in the Western Region, Ghana

**DOI:** 10.1186/s40795-023-00672-6

**Published:** 2023-01-10

**Authors:** Regina Enyonam Adonu, Millicent Amoah, Farrukh Ishaque Saah

**Affiliations:** 1 Department of Hospitality Management, Takoradi Technical University, Takoradi, Ghana; 2 Department of Epidemiology and Biostatistics, FN Binka School of Public Health, University of Health and Allied Sciences, Hohoe, Ghana; 3 Department of Population and Health, Faculty of Social Sciences, College of Humanities and Legal Studies, University of Cape Coast, Cape Coast, Ghana

**Keywords:** Breakfast intake, Barriers, Tertiary institution students, Western Region, Ghana

## Abstract

**Background:**

Breakfast is considered the day’s most important meal. Skipping breakfast consumption is detrimental to health and intellectual performance. University life has tight schedules and rigorous intellectual activities often very early in the morning. This study aimed at assessing breakfast intake and its associated factors among the students of Takoradi Technical University (TTU).

**Methods:**

This was a descriptive cross-sectional study. Data was collected from 347 students in TTU using pre-tested questionnaire. Data collected were analysed using STATA v17. It employed descriptive and inferential statistics such as logistic regression. *P*-value less than 0.05 was set as statistical significance at 95% confidence interval.

**Results:**

Regular breakfast was taken by 35.7% of the students. Higher odds of regular breakfast intake were found among respondents aged 25–29 years (AOR = 3.13, 95%CI = 1.57–6.24) and those who buy their breakfast (AOR = 5.13, 95%CI = 2.16–12.19). However, lower odds of regular breakfast consumption were found among respondents who were females (AOR = 0.40, 95%CI = 0.18–0.85). Barriers to regular breakfast intake included negative mood, insufficient funds, health condition, weight management, religious reasons such as fasting, limited time/unfavourable academic schedules, daily activities/workload, and cost of food on campus.

**Conclusion:**

The study stressed on the need for external and personal factors such as sex, age, religion, limited time/unfavourable academic schedules among others that hinder habitual breakfast intake to be addressed adopting innovative approach such as peer education and campaigns. University management should implement favourable policies on academic schedules, canteens/cafeterias, less stringent regulation on cooking at halls of residence.

## Introduction

The first meal of the day, breakfast, gives the brain the energy it needs to perform better cognitively. Poor cerebral glucose uptake may impair cognitive performance [1]. Breakfast is frequently referred to as the most crucial meal of the day and has been linked to good learning [2, 3]. A healthy lifestyle during youth and the early years of adulthood is heavily influenced by breakfast habits and intake [4, 5].

Several studies have shown that a regular meal schedule and eating breakfast can enhance daily diet quality and mental health [6–9]. Between supper and morning, the body uses the stored energy for around twelve hours [10]. During this time, the body consumes its reserves during the night, and the meal replenishes those reserves. The consumption of numerous nutrients, including vitamins A, E, C, B6, and B12, folate, iron, calcium, phosphorus, magnesium, and dietary fibre, is lower in people who miss breakfast compared to breakfast eaters. These deficiencies are seldom made up for at other meals [11–13].

By skipping breakfast during this fasting period, metabolic alterations can occur that interfere with some elements of cognitive function and academic achievement [14]. Missing breakfast may affect the availability of energy or certain nutrients required for the production of neurotransmitters, which are then required for proper central nervous system functioning [15]. Our brains are given energy and become more energized when we eat breakfast.

Individuals' eating habits are influenced by a wide range of variables, such as psychological, sociocultural, and educational aspects [16]. It is essential to determine who is more likely to miss breakfast and the causes of this behaviour [17]. According to prior research, kids with lower social and economic positions, less-educated parents, and parents who have divorced are more likely than other students to miss breakfast [16, 18]. Investigations revealed that students' lack of time in the morning, diminished appetite, and worries about their weight and appearance were the top causes of breakfast skipping [19, 20].

It is impossible to overstate the impact breakfast has on young people's health and performance, particularly university students. This is due to the fact that eating breakfast helps young people's attention, focus, intellectual aptitude, and ultimately academic success in addition to its relation to adequate nourishment [21, 22]. Despite the value of breakfast, many college students, especially those on Takoradi Technical University (TTU) campus, miss it frequently for a variety of reasons. Early morning (6:30am–9:30am) lectures at TTU were observed, and majority of the students reported skipping breakfast. This led to poor focus and diminished attentiveness. Thus, habitually missing breakfast is likely to be blamed for this lack of focus and inactivity during early morning lectures [21]. As several studies have demonstrated, if this keeps happening, academic performance may undoubtedly decline [17, 21, 22].

Due to the detrimental effects that poor breakfast intake behaviour has on students' cognition and performance, it is crucial to examine the breakfast intake behaviour of the students and its related components in order to design and implement particular solutions and treatments. So, in order to create a baseline of data for education and advocacy for reform, this study looked at Takoradi Technical University students' breakfast consumption and its associated characteristics.

## Methods

### Study design

This was a cross-sectional study collecting quantitative data from a section of university students in the Western Region of Ghana. The design allowed for selecting a representative sample from the population, quantifying breakfast intake, and determining statistical associations with the purpose of making inferences about the overall population [23].

### Study setting

The study was conducted at Takoradi Technical University in the Western Region of Ghana. It was founded in 1954. The University was formally a Polytechnic and was part of the first five to be upgraded into a Technical University in 2016. It has five faculties together with residential, recreational, market, and other non-academic facilities that cater to the needs of students and staff.

### Population

The study population was regular resident students of the Takoradi Technical University. This population has restricted time and a tight schedule, which affects their ability to care for themselves sufficiently. Only students living in university residences were included.

### Sampling

There were 1700 third-year students living in the five traditional halls of residence of the University as of July 2020, comprising: 622 in University Hall, 524 in GetFund Hall, 279 in Nzema Mensah Hall, 137 in Ahanta Hall, and 138 in Students Representative Council (SRC) Complex. The sample size determination table by Krejcie and Morgan was used to ascertain the sample size for the study, which recommends 313 samples for the population of 1700 [24]. A 10% non-response rate was calculated, and the final sample size was set at 347 students. Stratified proportionate sampling was used in selecting the study respondents. Each residential facility was considered a stratum, and each student in a residential facility had an equal chance of inclusion. At each facility, individual students were included until the allocated sample size was obtained.

### Procedures

Data were collected using pre-test questionnaires with the support of three research assistants. The research assistants were graduate students who are experienced in quantitative data collection and were given a day's training on the study instrument and purpose. The questionnaire comprised 18 items and was structured into four sections, Section A–D. Section A collected socio-demographic information of the respondents such as sex, age, religion, marital status, and source of and average income. While section B assessed breakfast intake behaviour (breakfast intake per week, time of consuming breakfast, source of food and food consumed for breakfast), section C focused on the assessing the barriers experienced by respondents in consuming breakfast regularly. The questionnaire comprised both open- and close-ended questions recorded a Cronbach alpha reliability score of 0.67 indicating having significant internal consistency. Data collection was carried out in the halls of residence, and students who consented were given the questionnaires to fill out. The questionnaires were thus self-administered but checked for validity and completeness after respondents filled them.

### Data analysis

The data collected were entered into a Statistical Package for Social Science (SPSS) template and exported to STATA version 17 for cleaning and analysis. The analyses included both descriptive and inferential statistics. Regular breakfast intake was determined by confirmation of daily breakfast consumption. The relationship between independent variables (namely, sex, age, religion, marital status, source of income, average monthly food expenditure, and breakfast meal source) and the dependent variable, breakfast intake. Statistical significance was set at a *p*-value less than 0.05 at a 95% confidence interval.

### Ethical issues

Ethical approval was given by the Institutional Review Board of the University of Education, Winneba, and permission was obtained from the management of the residential facilities. Before inclusion, written informed consent was obtained from students after the study purpose and procedure had been explained to them. To safeguard the anonymity and confidentiality of the study respondents, no personal identifying information was collected and the data collected has been kept under lock and key and soft copies stored on a password-protected personal computer of the researchers.

## Results

### Socio-demographic characteristics

As shown in Table 1, 347 students participated in the study with majority (53.6%) being females. Most of the respondents (63.1%) were aged 25–29 years while almost all (95.1%) were single. Again, majority (83.6%) were Christians whereas African Traditionalists were 1.7 percent. Most of the respondents (88.2%) had parents/guardians as source of income with relative majority (36.6%) having monthly expenses of GHS100-199.

**Table 1:** Socio-demographic characteristics of study respondents

Socio-demographic variable	Frequency (*N* = 347)	Percentage (%)
**Sex**		
Male	161	46.4
Female	186	53.6
**Age (in years)**		
< 20	21	6.1
20–24	107	30.8
25–29	219	63.1
**Marital status**		
Single	330	95.1
Married	17	4.9
**Religion**		
Christian	290	83.6
Islam	51	14.7
African Traditional	6	1.7
**Source of income**		
Parent/guardian	306	88.2
Self	41	11.8
**Monthly expenses (in GH₡)**		
< 100	98	28.2
100–199	127	36.6
200–299	93	26.8
300 +	29	8.4

### Breakfast intake among university students

**Fig. 1:**
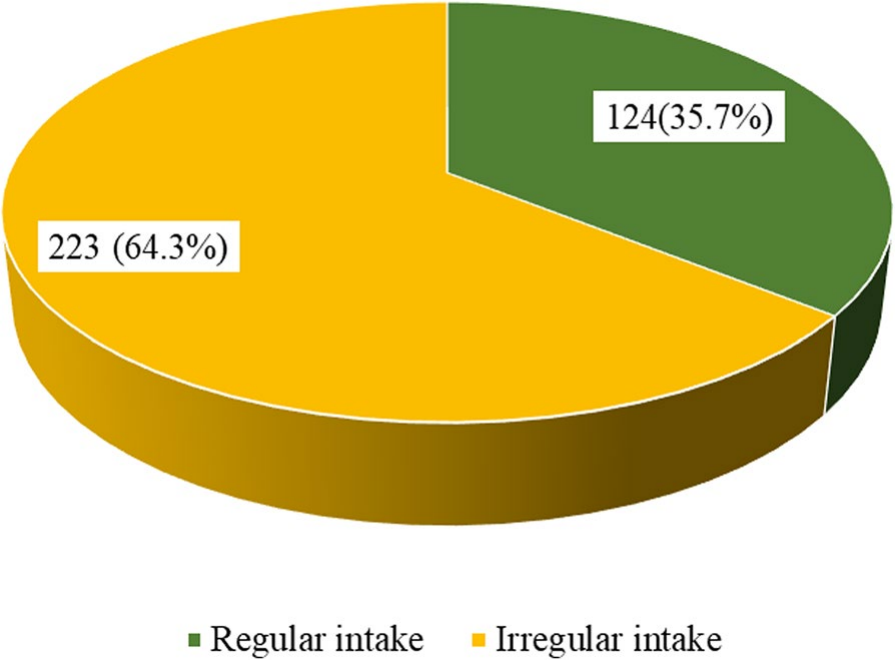
Prevalence of regular breakfast intake among students at TTU

Figure 1 presents the prevalence of regular breakfast intake among the University Students. It shows that less than half (35.7%) consume breakfast regularly.

Table 2 shows breakfast intake behaviour of the respondents. Only 35.7% take breakfast regularly with comparative majority consuming breakfast between 9-11am. Most of the respondents (71.8%) prepare their own breakfast.

**Table 2:** Breakfast intake behaviour among respondents

Breakfast intake variable	Frequency (*N* = 347)	Percentage (%)
**Breakfast intake**		
Always	124	35.7
Sometimes	223	64.3
**Times of breakfast intake**		
5am–7am	23	6.6
7am–9am	142	40.9
9am–11am	162	46.7
11am–1 pm	20	5.8
**Source of breakfast**		
Self-prepared	249	71.8
Buy it	98	28.2
**Typical meal for breakfast**		
Tea/milo beverage with bread	91	26.2
Porridge and bread/*Kose*	83	23.9
Rice with stew	69	19.9
*Waakye*	60	17.3
Other	44	12.7

### Factors associated with breakfast intake among university students

Table 3 shows the factors influencing breakfast intake among the study respondents. The chi square results shows that the students’ sex, age, religion, source of income, and source of breakfast were significantly associated with their breakfast intake. Following adjusting for significant variables, it was observed that females were 60 percent (AOR = 0.40, 95%CI = 0.18–0.85) less likely to take breakfast always than males. Students aged 25–29 years were 3.13 times (95%CI = 1.57–6.24) more likely to take breakfast always compared to those age less than 20 years old. Additionally, those who buy their breakfast compared to those who prepare their own breakfast were 5.13 times (95%CI = 2.16–12.19, *p* < 0.001) more likely to take breakfast always.

**Table 3:** Predictors of breakfast intake among respondents

Variable	Breakfast intake		*Χ*^2^(*p*-value)	COR(95%CI)*p*-value	AOR(95%CI)*p*-value
	**Always n(%)**	**Sometimes n(%)**			
**Sex**			4.59(0.032)*		
Male	48(29.8)	113(70.2)		Ref	Ref
Female	76(40.9)	110(59.1)		1.63(1.04–2.54)0.033*	0.40(0.19–0.84)0.016*
**Age**			16.49(< 0.001)***		
< 20	11(52.4)	10(47.6)		Ref	Ref
20–24	22(20.6)	85(79.4)		0.65(0.26–1.59)0.341	0.67(0.24–1.86)0.443
25–29	91(41.6)	128(58.4)		2.75(1.60–4.72) < 0.001***	3.13(1.57–6.24)0.001**
**Marital status**			2.30(0.129)		
Single	115(34.8)	215(65.2)			
Married	9(52.9)	8(47.1)			
**Religion**			10.23(0.006)**		
Christianity	95(32.8)	195(67.2)		Ref	
Islam	28(54.9)	23(45.1)		0.41(0.05–3.56)0.419	
African Traditional	1(16.7)	5(83.3)		0.16(0.02–1.51)0.110	
**Source of income**			6.50(0.011)*		
Parent/guardian	102(33.3)	204(66.7)		Ref	Ref
Self	22(53.7)	19(46.3)		2.32(1.20–4.47)0.012*	1.06(0.46–2.47)0.890
**Monthly food expenses (GH₡)**			3.42(0.332)		
< 100	37(37.8)	61(62.2)			
100–199	50(39.4)	77(60.6)			
200–299	26(28.0)	67(72.0)			
300 +	11(37.9)	18(62.1)			
**Source of breakfast**			27.26(< 0.001)***		
By self	68(27.3)	181(72.7)		Ref	Ref
Buy it	56(57.1)	42(42.9)		3.55(2.18–5.78) < 0.001***	5.03(2.43–10.40) < 0.001***

^*^p < 0.05

^**^p < 0.01

^***^p < 0.001

### Hindrances to regular breakfast intake among university students

Two main groups of barriers were found to hinder breakfast intake among the respondents who do not always take breakfast, as shown in Table 4. Personal barriers included mood (28.5%), insufficient funds (78.9%), health conditions (13.0%), weight management (19.5%), and religious reasons such as fasting (17.9%). Also, external factors include limited time and unfavourable academic schedules (92.3%), daily activities/workload (28.5%), and the cost of food on campus (14.2%).

**Table 4:** Barriers to breakfast intake among respondents

Hindrance	Frequency (*N* = 347)	Percentage (%)
**Personal barriers**		
Mood	70	28.5
Insufficient funds	194	78.9
Health condition	32	13.0
Weight management	48	19.5
Religious reasons	44	17.9
**External barriers**		
Limited time/unfavourable schedules	227	92.3
Daily activities or workload	70	28.5
Cost of food on campus	35	14.2

## Discussion

This cross-sectional study investigated breakfast intake and influencing factors among university students in Ghana, using Takoradi Technical University as a case study. It was discovered that students had a low regular breakfast intake. Regular breakfast intake was positively predicted by being aged 25–29 years old and buying one’s breakfast. It was, however, negatively predicted by being female. Individual’s mood, insufficient funds, an existing health condition, weight management, religious reasons (for example, fasting), and external factors such as limited time from unfavourable academic schedules, daily activities/workload, and the cost of food items on campus.

The study found that less than half of the respondents (35.7%) consume breakfast regularly. This finding is in congruence with those of Cebirbay et al. [10], who found that among the undergraduate students at a Turkish university, only 48.8% ate breakfast regularly. The result is similar to a previous study which observed that 34.8% of the students took breakfast regularly [25]. The proportion of regular breakfast intake found is also lower compared to that of Özdoğan et al. stating that 44.8% of the students regularly consumed breakfast [13]. However, this finding is lower than the findings of previous studies, which reported higher proportions of students who regularly take breakfast [17, 26]. Regular consumption of breakfast was practiced by 62% [17] and 54.5% [26] of the sample students in their studies. The observation could have resulted from the busy academic environment predominant at the Takoradi Technical University and the very limited access to eatery joints for breakfast due to the COVID-19 pandemic restrictions. This comes as the backdrop that location and easiness in choice and accessibility may facilitate and inhibit the habit of breakfast intake [14]. Poor accessibility may lead to skipping breakfast. This finding suggests that most students are not able to meet their nutritional needs every morning as they skip breakfast intake, and this has implications on their nutritional status, activeness, memory, and general performance during the early hours of the day.

Factors influencing regular breakfast intake were also identified by this study to include sex, age, religion, source of income, and source of breakfast. The current study found that respondents' sex predicted regular breakfast intake, which is consistent with many previous studies that found that females have higher odds of regular breakfast intake [12, 27–30]. This is consistent with several studies [31, 32] revealing that males are likely to skip breakfast compared to females even though Huang et al. [33] and Akarslan et al. [34] found otherwise. This thus, posits that greater focus should be given to male students to encourage habitual breakfast consumption.

Again, respondents’ age as a predictor of their regular intake of breakfast is consistent with the finding of Heo et al. [35], where the age of the respondents significantly influenced their intake of breakfast. This may suggest that older and more mature students have better control over their sources of income, their management, as well as their daily schedules, and thus are more organized and prepared to regularly take breakfast. This could also be attributed to a better understanding of the need for breakfast and taking cues from past experiences that have made them more willing and determined to consume breakfast regularly.

Again, the source of breakfast identified as a predictor of regular breakfast intake in this study backs up the findings of Ludin et al. [14], who discovered that access to breakfast had a significant relationship with students' ability to eat breakfast daily. This is consistent with the fact that due to a lack of time and possibly enough funds, many students are not able to prepare their breakfast at home/hostel and are thus, more likely to rely on some street foods such as porridge, which are cheaper and less time-consuming.

Also, barriers to regular breakfast intake included limited time/unfavourable academic schedules, a negative mood, insufficient funds, health conditions, weight management, religious reasons such as fasting, daily activities/workload, and the cost of food on campus. With regards to limited time being a barrier to regular breakfast intake, this supports previous studies that also observed that lack of time in the morning usually barred students from regular breakfast intake [10, 17, 18, 20].

In addition, weight management has also been reported in a similar study where some students indicated that they skip breakfast for concerns about their body image and weight [20]. Insufficient funds and cost of food on campus as factors barring students from regular breakfast consumption is in congruence to the findings of Wijtzes et al. [16] in a related study. The researchers found that lower social economic status of students affects their ability to afford or prepare breakfast regularly and this is in congruence with Garrido-Fernández et al.’s finding that money management hinder healthy eating habits of young people [19]. Additionally, negative mood, including reduced appetite and not feeling hungry has been identified in previous studies [17, 20, 25].

More so, heavy academic workloads and schedules lead to stress, which makes some students either wake up late or arrive late for school, as reported by Yaman and Yabanci [25]. These observations could have resulted from the fact that most of the students in this study have parents/guardians as their main source of funds, which affects their ability to spend adequately to cover regular breakfast. Also, these observations could be attributed to the tighter schedules and increased academic work associated with final-year studies, as all the respondents were in their final levels in their programmes. This is consistent with the finding by Garrido-Fernández et al. that most schools are not promoters of healthy eating habits [18].

Another barrier to habitual breakfast consumption found was religion, which is in congruence with Danquah et al.’s study in Ghana, noting that religion has a significant impact on people’s consumption of breakfast [31]. Religious activities such as fasting change eating patterns as well as influence one’s ability to eat breakfast regularly. Heo et al. reported that income, including its source and amount, influences one’s ability to afford a meal either by preparing one or buying outside the residence [35].

### Strengths and limitations

This study went beyond simple verbal report of eating breakfast or not to include the timing, foods, and regularity of breakfast intake. Furthermore, it is astounding how little research has been done to promote healthy eating among tertiary students. Thus, the study might be a novelty that inspires further action. However, a major limitation of the study is its reliance on a verbal report by the respondents. This has the potential of over-reporting or under-reporting social behaviours such as breakfast intake. However, the respondents were encouraged to provide honest responses as much as possible. Also, due to the COVID-19 preventive measures, some students were not available on campus during the period of data collection. These included first-year students.

## Conclusion

The majority of the students are not taking breakfast, which may negatively impact their memory, activeness, performance, and ability to meet the required nutrient value for rigorous academic work. Should this situation persist, most students may become malnourished or perform poorly due to poor memory capacity resulting from inactivity in class and poor concentration from skipping breakfast. The current observation has grave implications for the poor well-being and academic performance of the students. The environmental factors, family background, variety of choices, and accessibility in terms of location and ease of getting the food were associated with breakfast consumption. Thus, it is suggested that measures such as readjustment of the school timetable, reducing the cost of food, establishing canteens/restaurants within halls/hostels, and removal of a strict set of hall rules and regulations restricting the use of cooking utensils and gas cookers should be implemented by the management of universities.

## Data Availability

Dataset for the study can be obtained upon request from the corresponding author.
